# Role of Molecular Epidemiology on Tuberculosis Control in the Middle East Countries: a Systematic Review and Meta-Analysis

**Published:** 2018-10

**Authors:** Mahya Pourostadi, Jalil Rashedi, Behroz Mahdavi Poor, Hossein Samadi Kafil, Abdolhassan Kazemi, Ehsan Ahmadpour, Mohammad Asgharzadeh

**Affiliations:** 1 Hematology and Oncology Research Center, Tabriz University of Medical Sciences, Tabriz, Iran,; 2 Tuberculosis and Lung Diseases Research Center, and Department of Laboratory Science, Faculty of Paramedicine, Tabriz University of Medical Sciences, Tabriz, Iran,; 3 Department of Laboratory Science, Faculty of Paramedicine, Tabriz University of Medical Sciences, Tabriz, and Department of Medical Parasitology, School of Medical Sciences, Tarbiat Modares University, Tehran, Iran.,; 4 Drug Applied Research Center, Tabriz University of Medical Sciences, Tabriz, Iran.,; 5 Infectious and Tropical Diseases Research Center, Tabriz University of Medical Sciences, Tabriz, Iran.,; 6 Department of Parasitology, Faculty of Medicine, Tabriz University of Medical Sciences, Tabriz, Iran.,; 7 Biotechnology Research Center, Department of Laboratory Science, Faculty of Paramedicine, Tabriz University of Medical Sciences, Tabriz, Iran.

**Keywords:** Molecular epidemiology, MIRU-VNTR, tuberculosis, transmission, Middle East

## Abstract

**Background:**

Tuberculosis (TB) is a public health problem in developing countries and yet the numbers of people with the disease are abundant. Early detection of transmission sources and effective treatment of the cases is essential to control the disease which will be possible by application of molecular epidemiology approaches. Studies conducted based on Mycobacterial Interspersed Repetitive Units-Variable Number of Tandem Repeats (MIRU-VNTR) method in Muslim Middle East countries were evaluated to determine their role in TB control.

**Materials and Methods:**

All studies from January 2005 to April 2016 were systematically reviewed in four electronic databases and finally 16 articles were found eligible to be included in this study. The mean clustering rate was determined as 44% and the recent transmission rate was 12.3 to 78.8% with average of 33%.

**Results:**

The results showed that both reactivation and recent transmission were important in developing new cases of TB in Middle East countries; but, reactivation plays a more critical role.

**Conclusion:**

Regarding to ongoing war and immigration in the region along with the increasing of drug-resistant TB, in the case of improper supervision in the future, the disease, especially drug- resistant TB, will be problematic.

## INTRODUCTION

Throughout history, *Mycobacterium tuberculosis* (*M. tuberculosis*) has caused the highest mortality (1). At the moment, approximately one- third of the world’s population is infected with the bacteria (2). According to WHO report, 9.6 million new Tuberculosis (TB) cases occurred all around the world in 2014 of which 1.5 million died from them, 190,000 deaths were due to MultiDrug- Resistant TB (MDR-TB) and 400,000 cases of them were co-infected with Human Immunodeficiency Virus (HIV) (3). At present, drug-resistant *M. tuberculosis* strains with growing rate of prevalence in many countries are a serious threat for human beings (4). Planning for TB control needs to identify the sources of infection (5), the transmission trend (6), and factors affecting treatment failure, so that the spread of the disease will be prevented by prohibiting the transmission of the infection.

Increasing the knowledge of researchers about the genome of the bacteria, makes it possible to identify the various strains of the *M. tuberculosis* (7). Genotyping of *M. tuberculosis* isolates from patients in a region, lead to determination of dominant strains (8), transmission pattern (9), and associated risk factors (10). The results of genotyping studies can be used for establishing the disease control measures.

Several molecular typing methods are being used in molecular epidemiology studies and genotyping (11). One of these methods is Mycobacterial Interspersed Repetitive Units-Variable Number of Tandem Repeats (MIRUVNTR), in which repetitive units that are dispersed on the mycobacterial genome are used to study. Each unit comprises of 40–100 bp and various numbers of the units have been scattered as tandem repeats in the M. tuberculosis complex genome. Overall, there are 41 MIRU loci that 12, 15, and 24 loci are used for genotyping of the bacteria (12,13).

Various strains of *M. tuberculosis* contain different number of repeats in different positions and the repeats are detectable by Polymerase Chain Reaction (PCR). The repeats can be calculated based on the size of amplified products (14). MIRU-VNTR method is considered as an alternative to the IS6110-RFLP for simplicity and high precision of it and today it is used for epidemiological studies.

In this study, the researches conducted based on the MIRU-VNTR in Muslim Middle East countries were systematically reviewed, to determine the molecular epidemiological studies role in TB control and this may help to make preventive decisions in decreasing the rate of the disease in the region.

## MATERIALS AND METHODS

### Study selection

This research consisted of molecular epidemiology studies on Middle East Muslim populations. All studies that had the following characteristics and information were included in this study: using the MIRU-VNTR method as primary method for genotyping, the number of examined cases more than 17, the study period of 6 months and above, and the determined numbers of isolates within each cluster. The researches that had used methods other than MIRU-VNTR as primary ones, study on non- *M. tuberculosis* strains or non- human samples were excluded from this study.

### Literature search

Four electronic databases consisting of Google scholar, Scopus, PubMed, and Scientific Information Database were examined from 2005 to April 2016. The search was done by using the keywords of tuberculosis, MIRU, and Middle East to find all English articles about these topics.

### Data extraction

The collected data included the time of the study, the duration, sample size, country, city or region, genotyping method, the secondary typing method, number of isolates within each cluster, cluster size, cluster numbers, isolates with unique pattern, clustering rate, recent transmission rate, and associated risk factors of the studies. To calculate the clustering rate the formula (15) below was used, assuming that each cluster has an infected source in which the disease has become active and the others members of the cluster have acquired the disease from that source recently.

Minimum estimated rate of recent transmission =

$$\frac{\text{Number}\;\text{of}\;\text{clustered}\;\text{patients}-\text{Number}\;\text{of}\;\text{clusters}}{\text{Total}\;\text{number}\;\text{of}\;\text{patients}}$$

The isolates which had unique pattern in the number of repeats were categorized as non- clustered and the strains which had the same MIRU-VNTR patterns were classified as clustered.

## RESULTS

**Figure 1.**
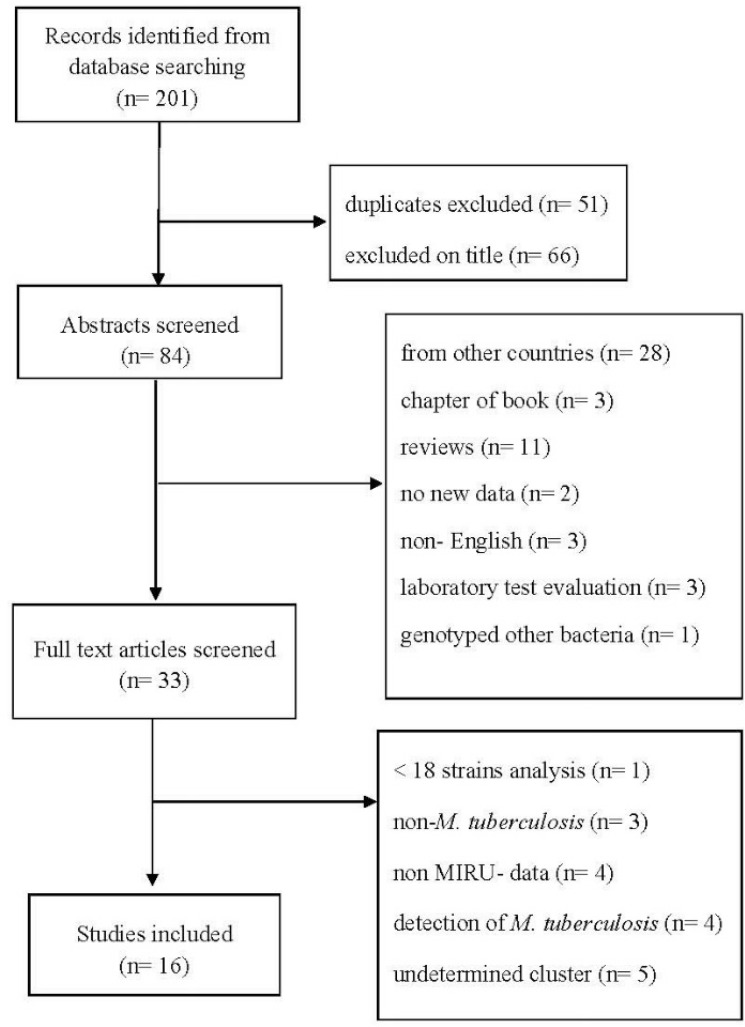
Flow diagram of literature review process.

The published papers related to the genotyping of *M. tuberculosis* strains were reviewed and those which had not conducted molecular analysis or used methods other than MIRU-VNTR, or whether they had not mentioned number of isolates within each cluster were excluded from the study. Finally, the data from 16 articles were examined (Figure 1).

**Figure 2.**
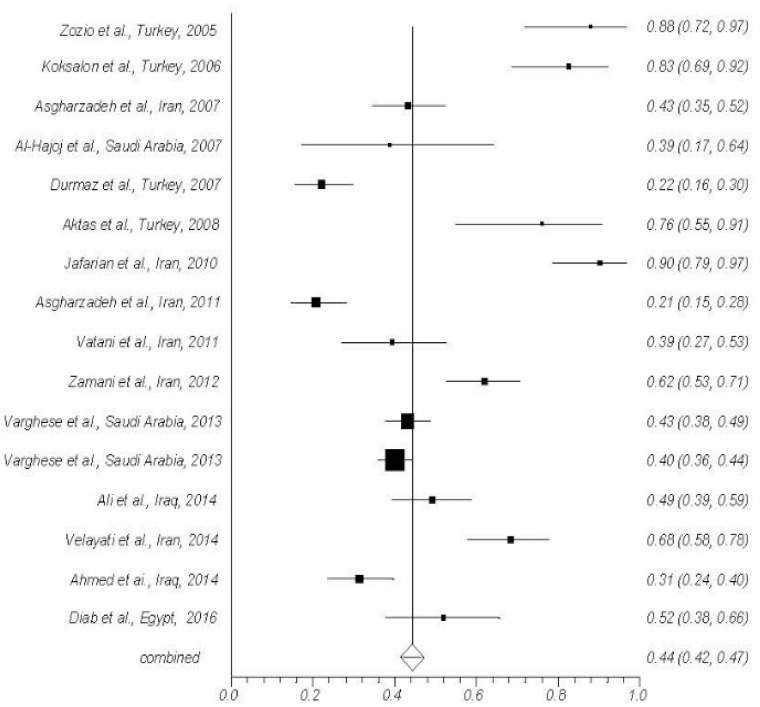
Forest plot of clustering rate in 16 studies (proportion with 95% confidence interval).

Essential data extracted from the articles are summarized in table 1 (16–31). In thirteen studies secondary fingerprinting methods such as IS6110-RFLP and spoligotyping were used and in three studies the secondary fingerprinting methods were not applied (22, 23, 29). All cases caused by lab contamination and non- *M. tuberculosis* infection, were excluded in the sample studies. The average rate of clustering was 44% (Figure 2). The maximum number of isolates within a cluster was 24, which had been in Saudi Arabia (20). The maximum clustering rate was seen in Tehran (90.2%), the capital city of Iran (25). In reviewed studies the average rate of recent transmission was 33%. The highest rate of recent transmission was 78.8% [(29-3)/33] which had been in Malatya and Ankara, Turkey (31). In Saudi Arabia being young was a risk factor (P<0.05) (20) and in Zonguldak, Turkey being young and middle-aged (P= 0.006), and drug resistance (P= 0.02) were risk factors (26). In East and West Azarbaijan provinces, north western part of Iran, individuals with age above 56 years were mostly within the cluster (P=0.31) (24).

**Table 1.** Selected characteristics of included studies

**No**	**First author (reference)**	**Study region**	**Year of publication**	**Study years**	**Study duration of months**	**Secondary Genotyping method**	**Study subjects**	**Unique patterns**	**Isolated included in clusters**	**No. of clusters**	**Cluster size**	**Clustering Rate %**	**Recent transmission %**
1	Diab (16)	Egypt	2016	12–14	24	spoligotyping	52	25	27	10	2–6	52	32.7
2	Ahmed (17)	Iraq	2014	11–12	18	spoligotyping	134	92	42	8	2–8	31.3	25.4
3	Velayati (18)	Iran	2014	10–11	12	spoligotyping	92	29	63	23	2–5	68.5	43.5
4	Mustafa Ali (19)	Baghdad, Iraq	2014	10–11	13	spoligotyping	110	56	54	17	2–9	49.1	33.6
5	Varghese (20)	Saudi Arabia	2013	09–11	24	spoligotyping	524	314	210	42	2–24	40.2	32.1
6	Varghese (21)	Saudi Arabia	2013	09–10	12	spoligotyping	322	183	139	37	2–16	43.2	31.7
7	Zamani (22)	Iran	2012	10	12	-	121	46	75	20	2–13	62	45
8	Vatani (23)	Khuzestan, Iran	2011	08–10	21	-	61	37	24	11	2–3	39.3	21.3
9	Asgharzadeh(24)	East and westAzarbaijan	2011	04–05	12	IS6110-RFLP	154	122	32	13	2–6	20.7	12.3
10	Jafarian (25)	Tehran, Iran	2010	09	12	spoligotyping	51	5	46	7	2–8	90.2	76.5
11	Aktas (26)	Zonquldak, Turkey	2008	03–05	36	spoligotyping	25	6	19	1	19	76	72
12	Durmaz (27)	Malatya, Turkey	2007	00–04	60	IS6110-RFLP, spoligotyping	145	113	32	13	2–7	22	13.1
13	Al-Hajoj(28)	Saudi Arabia	2007	02–05	36	spoligotyping	18	11	7	2	3–4	38.9	27.8
14	Asgharzadeh(29)	East Azarbaijan, Iran	2007	02–03	6	-	127	72	55	21	2–6	43.3	26.8
15	Koksalon (30)	Istanbul,Turkey	2006	02–04	30	spoligotyping	46	8	38	6	2–19	82.6	69.6
16	Zozio (31)	Malatya andAnkara, Turkey	2005	98-04	72	spoligotyping	33	4	29	3	2, 8, 19	87.9	78.8

## DISCUSSION

Nowadays, using DNA genotyping approaches makes it possible to assess the TB control programs and determine the recent transmission rate, so that in 16 reviewed studies based on MIRU-VNTR in Muslim Middle East countries, the recent transmission rate was between 12.3 to 78.8%, with average of 33% (892- 234/ 2015). Overall results showed that majority of TB cases in the countries were due to reactivation (67%) rather than recent transmission. According to the obtained results, the recent transmission rate varies in different countries and times. So that in studies conducted before 2011, despite the low number of isolates, majority of the cases were due to the recent transmission (25, 26, 30, 31). While, studies conducted later indicated the decreasing transmission rate, so that majority of evaluated isolates had unique pattern (16–24). Therefore, most of the cases were due to reactivation that was in agreement with decreasing rate of TB prevalence in the countries.

In half of the reviewed studies the numbers of evaluated samples with MIRU-VNTR method was less than 100 cases. Hence, this could possibly lead to bias in determining the recent transmission rate. Fortunately, the later studies conducted with enough sample size and obtained results were reliable. The average 33% recent transmission rate in our studied countries is closer to the study result conducted in Casablanca, Morocco (37%) (32), but is higher than Stockholm study (13.3%) (33) and is lower than Xi’an, China (60%) (34). The high recent transmission rate in China study was probably due to the prevalence of Beijing strain which is a high transmissible strain (35,36). Therefore, the improvement of TB control strategies and use of molecular genotyping methods may have been effective in decreasing the number of TB cases in China (3).

The average rate of clustering among the reviewed studies was 44 %. The lowest clustering rate was reported 20.7% in East and West Azarbaijan provinces from north western part of Iran by Asgharzadeh et al. (24). While, the highest rate was observed 90.2 and 87.9% in Tehran, the capital city of Iran, by Jafarian et al. (25), and Malatya and Ankara, Turkey (31), respectively. The clustering rate are affected by some factors for instance: age of the subjects (37), being new case (38), local TB incidence, and existence of special strains such as Beijing (39). In north western part of Iran, the clustering rate was low. This was probably because of the very low numbers of immigrants from other countries, especially from Afghanistan, and consideration of control measures in young people. On the other hand, the age of most subjects was above 60 years that led to reactivation of more latent TB infections (24). Meanwhile, in Malatya and Ankara, Turkey, the higher frequency of certain strains had increased the clustering rate in the examined samples (31).

In the conducted studies, the most common cluster size was 2, and the largest cluster size was reported 24 by Varghese et al. (20), in Saudi Arabia. This study was done with adequate sample size (524 isolates) during 24 months. Adequate sample size and relatively appropriate duration of the study lead to an increase in the cluster size. However, in some studies ( 16 , 18 , 23 , 28 ), the largest cluster size due to low number of subjects was smaller than Saudi Arabia (20). But, in Stockholm, Sweden, despite the proper sample size, the largest cluster had only 4 members (33) and in researches carried out in Victoria, Australia (40), Hong Kong (41), and Samara, Russia (39) the largest clusters contained 95,77, and 75, respectively, that was due to the existence of Beijing strain with considerable transmission power (35).

One of the most important factors in increasing the size of the cluster is the raising of the specific strains in an area (42). Regarding that the specific strain does not have considerable frequency in the Middle East countries; therefore, the clusters size was relatively small. So, it can be concluded that the occurrence of the TB in the countries is micro-epidemic. In addition, in some of the studies sample size and duration were relatively low that lead to the majority of cases remained unrecognized. Also, in the surveyed countries, in total, the population of alcohol consumer, intravenous drug abuser, homeless people, and HIV infected individuals are low; therefore, in the reviewed studies the cluster sizes were small.

In term of discriminatory power MIRU-VNTR followed by IS6110-RFLP are the most sensitive methods for *M. tuberculosis* genotyping. In MIRU-VNTR method, with increase in the numbers of evaluated loci, discriminatory power will be increased and even can be more powerful than IS6110-RFLP. IS6110- RFLP is a time consuming method and in case of 5 or less than 5 IS6110 bands, its discriminatory power is also low (43). Other genotyping methods with relatively lower discriminatory power include: Single Nucleotide Polymorphism (SNP) (44), spoligotyping, direct repetitive element- PCR (DREPCR), and Pulsed-Field Gel Electrophoresis (PFGE) (45).

In order to obtain reliable results in SNP analysis, the large number of genes must be investigated. In PFGE method the bacteria whole DNA molecule is needed for cutting by enzyme. Despite of spoligotyping approach is the ideal method for detecting Beijing strain; it has low discriminatory power (33). Using spoligotyping method exclusively enables it to estimate the recent transmission rate and it should be used with a secondary typing method for genotyping.

In addition, to determine the recent transmission rate, MIRU-VNTR method is capable to detect laboratory cross-contamination (46), nosocomial transmission, mixed infection (47), and recurrent TB caused by reinfection. In regions with high prevalence rate of TB, more people are exposed to *M. tuberculosis*, therefore, it leads to increasing number of reinfection cases (48). Furthermore, in susceptible individuals such as immunocompromised ones the reinfection rate is high (49).

TB disease manifests clinically in limited number of infected persons. Several factors including the infectious agent, host and environmental properties are effective in catching the disease (50). Some risk factors are as follows: being imprisoned, drug abuse, alcohol consumption, homelessness (51), infection with HIV (15), and immigration (52). Immigrants play a significant role in TB epidemiology in different populations. In Western European and Scandinavian countries TB transmission has occurred mainly among the immigrant groups and their children (53,54). The majority of TB cases in Iran (55), and Saudi Arabia (20,21) were also among immigrant populations. This is due to the fact that immigrants live in small and crowded places and are exposed to inadequate facilities, malnutrition, stress (56), and poor sanitation (57). Therefore, they are at high risk of afflicting TB disease. On the other hand, reactivation of latent infection in the population may create a source for transmission to other persons (58). The reactivated cases may import clinically dangerous isolates to the host country. Thus, it is necessary to identify latent infections and treatment of the disease among immigrant population to prevent the increase of MDR-TB cases in the host countries.

Being young is also the other risk factor (38). Among reviewed studies, in Saudi Arabia (20) and Turkey (26) being young was a risk factor. In contrast, in Asgharzadeh et al. study (24) which was conducted in two provinces of northwest of Iran, TB was more prevalent among persons above 56 years of age. This issue was more likely due to poor living condition, low literacy, inadequate income, and food poverty among elderly people. Also, it could be due to low prevalence of HIV infection and appropriate TB control performance in young people and even success in controlling TB infection in young people in this area of Iran.

The other risk factor is drug- resistance (4). In Belgrade (59), Estonia (4), Archangel Oblast, Russia (60), and Zonguldak, Turkey drug-resistance was risk factor (P<0.02) (26). In Belgrade due to insufficient TB control plans, drug- resistance was increased (59). In Estonia (4), and Archangel Oblast, Russia (60) the high frequency of Beijing strain had the major role; while, in Turkey the frequency of Beijing strain was low (61).

In recent years, HIV infection has been enhanced in Middle East countries (3). Considering the high susceptibility of HIV positive individuals to acquire TB infection, it is necessary to make the people aware, especially the youth, about the HIV transmission routes for decreasing TB prevalence.

The reviewed studies had some limitations which are as follows: Different typing methods had been applied in the surveys; the duration of some of the studies was less than 18 months; in some of the researches risk factors were evaluated, but the factors were different; in some studies only positive- culture cases were included, and pediatrics TB cases which are usually negative culture due to the lack of proper sample preparation, were missed. Surprisingly, there were cases that had not any contact with TB patients, but were within the clusters.

It is recommended that at least MDR-TB isolates in Middle East countries be collected and evaluated using 24 MIRU loci to identify the majority of existing drug- resistant strains in the countries. By identifying the drug-resistant strains and interrupting the transmission cycle, the development of extensively drug- resistant tuberculosis (XDR-TB) could be prevented.

## CONCLUSION

Molecular epidemiologic studies by using MIRU- VNTR with determining the recent transmission rate and effective factors in Middle East countries make it possible to assess the TB control program. Furthermore, studies have revealed that new cases of TB, both reactivation and recent transmission, are important in countries, however, reactivation plays a more significant role.
